# Intravascular Hemolysis and Acute Kidney Injury Following an Emergent Cesarean Delivery: A Case Report

**DOI:** 10.7759/cureus.90331

**Published:** 2025-08-17

**Authors:** Sanjana D Nalla, Sejal Chopra, Sreedevi Rama, Constantino G Lambroussis

**Affiliations:** 1 Obstetrics and Gynecology, Lifeline Medical Associates, Edison, USA; 2 School of Medicine, Lake Erie College of Osteopathic Medicine, Erie, USA; 3 Obstetrics and Gynecology, Saint Peter’s University Hospital, New Brunswick, USA; 4 Osteopathic Medicine/Family Medicine, Lake Erie College of Osteopathic Medicine, Elmira, USA

**Keywords:** acute kidney injury, cesarean section, multidisciplinary management, obstetric complication, oliguria, postpartum hemolysis

## Abstract

The uncommon occurrence of intravascular hemolysis leading to acute kidney injury (AKI) in the postpartum period, particularly following an emergent cesarean section (C-section), represents a serious complication that requires prompt recognition and management. This report highlights the case of a 31-year-old woman at 37.4 weeks of gestation who underwent an emergency C-section due to persistent fetal bradycardia. Her immediate postoperative course was complicated by oliguria, a drop in hemoglobin, and signs of hemolysis, such as elevated lactate dehydrogenase (LDH), low haptoglobin, low hemoglobin, and elevated bilirubin levels. These laboratory findings were suggestive of intravascular hemolysis, which, when coupled with AKI, can be life-threatening without early intervention. Our case emphasizes the importance of early identification and a multidisciplinary approach in managing postpartum intravascular hemolysis and AKI to optimize maternal outcomes.

## Introduction

Hemolytic anemia, a condition in which red blood cells (RBCs) are prematurely destroyed, is a complication that can occur during pregnancy with implications for both the mother and baby. While the etiology is multifactorial, the pathophysiology in pregnancy-related hemolysis includes infections, acute liver adiposal dystrophy, amniotic fluid embolism (AFE) leading to disseminated intravascular coagulation (DIC), and hemolysis, elevated liver enzymes, and low platelet count (Hemolysis, Elevated Liver enzymes and Low Platelets (HELLP) syndrome) due to underlying preeclampsia. Autoimmune etiologies, such as autoimmune hemolytic anemia, are also notable causes of hemolysis, leading to severe maternal anemia, with an average occurrence of 1-3 per 100,000 cases per year [1]. Hemolysis can lead to significant maternal complications, such as delayed postpartum recovery and organ dysfunction, among others; therefore, comprehensive management is essential [1].

Acute kidney injury (AKI) is defined as a sudden decline in renal function, leading to disturbances in electrolyte balance, fluid build-up, and even multiorgan system dysfunction. In pregnancy, the development of AKI can arise from various clinical presentations, including hyperemesis gravidarum, septic abortion, and preeclampsia [2]. Once a patient is diagnosed with AKI at any point in the course of the pregnancy, the risk for maternal and fetal morbidity, such as cardiovascular events and longer hospital stays, is higher. Efficient diagnosis is crucial for effective management, as the incidence of pregnancy-related AKI in the United States has been shown to increase from 0.04% in 2006 to 0.12% in 2015 [3].

The co-existence of hemolytic anemia and AKI during pregnancy creates a clinically complex situation that requires a thorough investigation of all potential causes. However, early identification and treatment are crucial for an unknown etiology when a patient exhibits no more compelling evidence for the prior diagnoses mentioned. Further research is necessary to fully understand the relationship between hemolytic anemia and AKI and how they arise during pregnancy. This is essential as the overlap with normal physiological changes during pregnancy can mask any early symptoms and raise the patient's risk of adverse effects [4]. For example, parameters such as decreased hemoglobin/hematocrit, mild elevations in lactate dehydrogenase (LDH), and lower platelet count are all shared features of normal pregnancy, AKI, and hemolytic anemia, making the ruling-out process challenging. 

## Case presentation

A 31-year-old woman, G3P0111, at 37 weeks and 4 days of gestation, presented to the hospital’s labor and delivery unit with concerns of decreased fetal movements, fluid leakage, and contractions every 30 minutes. The review of systems was otherwise negative. Her medical history was remarkable for a prior induced abortion and a subsequent pregnancy, which resulted in a preterm vaginal delivery due to premature rupture of membranes (PROM) at 32 weeks and 2 days. No other medical issues or family history were recorded. The patient’s body mass index (BMI) at the pregnancy confirmation visit was 28.4. Her vital signs were within normal limits, with the physical examination showing no pooling or ferning. The cervix was closed with a Bishop score of 0. A non-stress test (NST) was reactive with occasional contractions, and the patient reported fetal movements. With no additional concerns at that time, she was discharged home with appropriate instructions for follow-up.

Regarding the current pregnancy, a routine urine culture at 13 weeks revealed *Enterococcus faecalis*, which was successfully treated with a seven-day course of Macrobid (nitrofurantoin 100 mg twice daily) with no recurrence. Given the patient’s history of preterm delivery in a previous pregnancy, starting at 17 weeks, she was managed with weekly 17-hydroxyprogesterone injections and daily Aspirin 81 mg. Her 20-week fetal anatomy scan revealed marginal placenta previa, which resolved by 30 weeks, as confirmed by follow-up ultrasound. Prenatal laboratory tests, including a comprehensive obstetric panel and an oral glucose tolerance test, were within normal limits. Infectious disease screening was negative for human immunodeficiency virus (HIV), hepatitis B surface antigen, syphilis via rapid plasma reagin (RPR), and Rubella. There was no clinical or laboratory evidence of gestational hypertension or preeclampsia, and the patient denied experiencing headaches, blurred vision, nausea, or vomiting throughout the current pregnancy.

Within a few hours of discharge, the patient returned to the hospital with excruciating abdominal pain and uncertainty about whether she was experiencing contractions. Upon evaluation, fetal bradycardia was noted, with the fetal heart rate (FHR) persistently in the 70s. Terbutaline was administered in an attempt to reduce tetanic uterine contractions, but the fetal bradycardia persisted. A STAT C-section was performed under general anesthesia. The infant was delivered atraumatically, weighing 6 lbs 10 oz (3005 g). The scores of appearance, pulse, grimace, activity, and respiration (APGAR) were 2 and 7 at 1 and 5 minutes, respectively. The newborn was initially admitted to the neonatal intensive care unit (NICU) but was promptly returned to the mother. The quantified blood loss during the cesarean section (C-section) was 487 mL, and the intraoperative urine output was 75 mL. Upon awakening from general anesthesia, the patient exhibited signs of agitation and combativeness. She attempted to remove her IV lines and oxygen mask, necessitating physical restraint by the operating room team. The patient was subsequently stabilized and transferred to the recovery room. She was noted to have steady improvement in alertness, orientation, and speech/thought, decreasing the suspicion of delirium. 

Postoperatively, minimal urine output was observed through the patient’s Foley catheter. Despite receiving 5 L of lactated Ringer's solution, which included a 3 L IV bolus and 1500 mL intraoperatively, the patient’s urine output immediately after surgery was 60 mL, with no notable urine output for nine hours. A tense 6 cm x 3 cm subcutaneous, non-expanding hematoma was palpated near the right side of the C-section incision site, which was tender and had slight oozing of blood from the incision site. There was no significant vaginal bleeding, and a CT scan of the abdomen and pelvis ruled out intra-abdominal bleeding. Maternal-Fetal Medicine (MFM) was consulted due to concern for potential AKI and DIC. The Foley catheter was flushed and replaced twice, and a subsequent bladder scan was empty. The patient’s vital signs remained stable.

**Figure 1:**
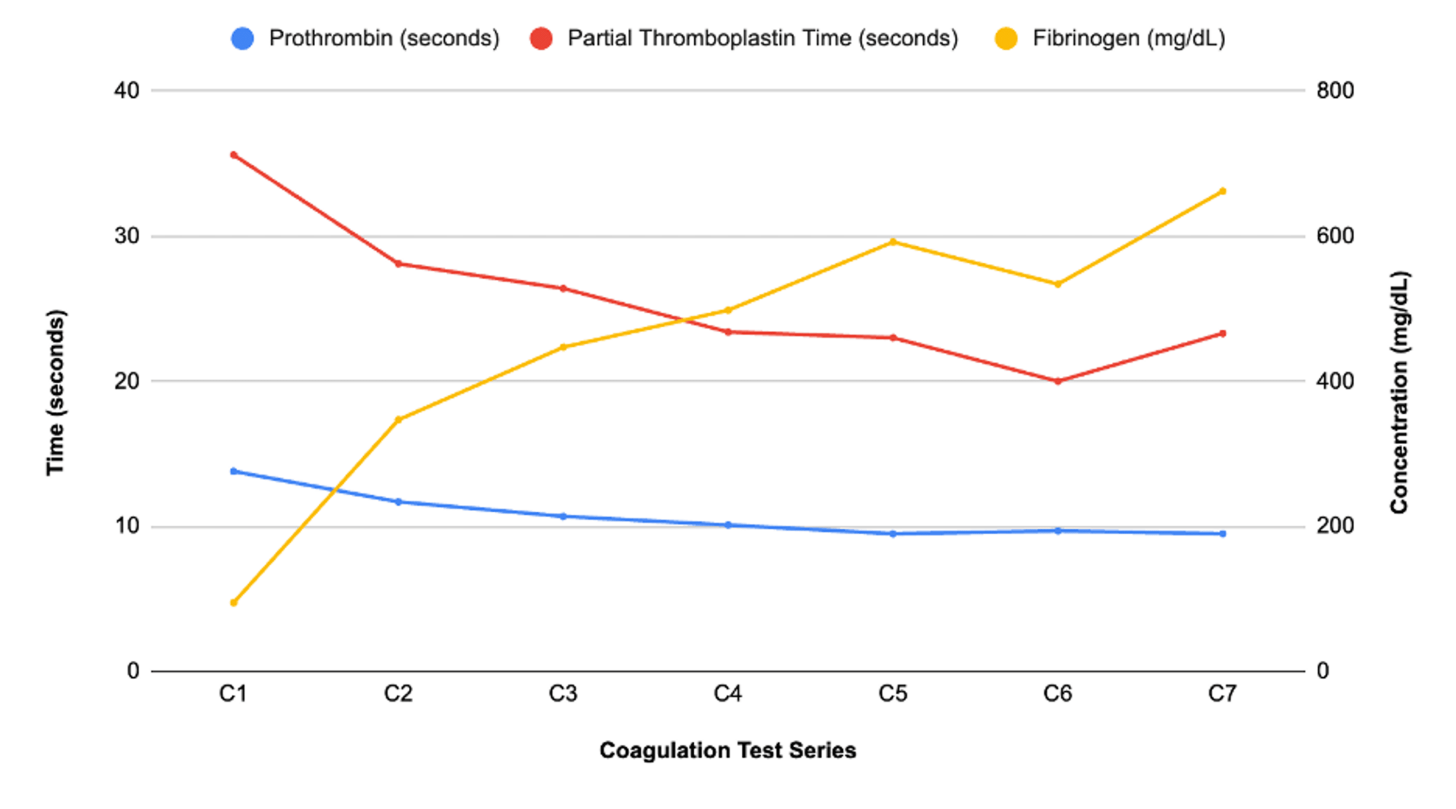
Coagulation profile trends during the patient’s hospitalization. Values represent PT, PTT, and fibrinogen concentrations, labeled C1 through C7. All values were obtained during the patient’s hospitalization. PT: prothrombin time; PTT: partial thromboplastin time.

**Figure 2:**
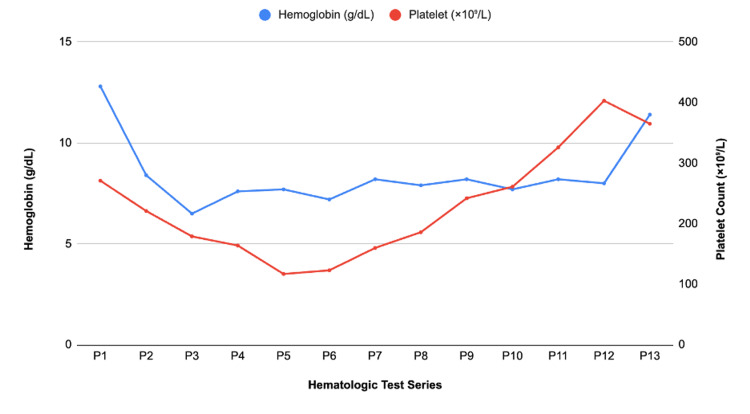
Patient’s hematologic trends during hospitalization and the postpartum period. Values represent hemoglobin and platelet measurements labeled P1 through P13. P1–P11 were obtained during hospitalization. P12 and P13 were obtained during outpatient postpartum follow-up visits at 10 days and 52 days postoperatively, respectively. A drop in hemoglobin to 8.4 g/dL postoperatively on P2 from >10 g/dL preoperatively raised concern for potential hemolytic anemia, prompting workup.

When attempting to obtain STAT labs, the samples were hemolyzed twice, raising the concern for intravascular hemolysis. Following the two hemolyzed samples, a complete blood count (CBC), comprehensive metabolic panel (CMP), coagulation studies (shown in Figure 1), and a type and cross were drawn. Given the concern for AKI due to acute blood loss and third spacing versus HELLP syndrome or DIC, a recommendation for transfer to the intensive care unit (ICU) and further evaluation was made. In the ICU, labs revealed a hemoglobin drop from 12.8 pre-op to 8.4 post-op, and a stable platelet count. Figure 2 shows hemoglobin and platelet measurements in our patient. The international normalized ratio (INR) was mildly elevated, making DIC a less likely etiology. The patient was transfused with one packed red blood cell unit and two fibrinogen units.

**Figure 3:**
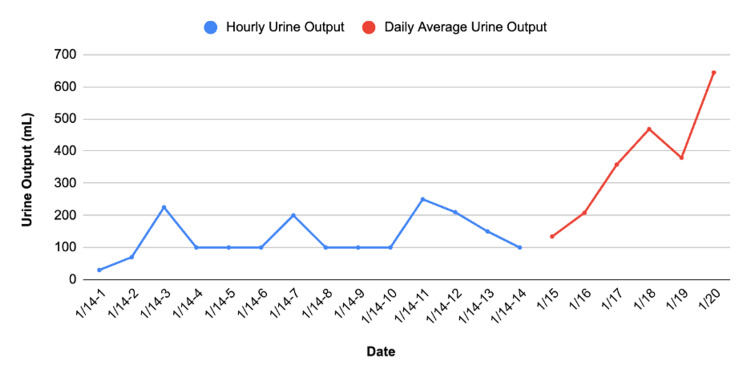
Trend of the patient’s immediate postoperative hourly urine output (1/14/25) and daily average urine output from 1/15/25 to the day of discharge (1/20/25). The initial hourly data reflect close monitoring after the C-section, while the subsequent daily urine output averages demonstrate progressive renal function improvement. The most notable improvement was noted from the day of discharge into the following few days.

**Figure 4:**
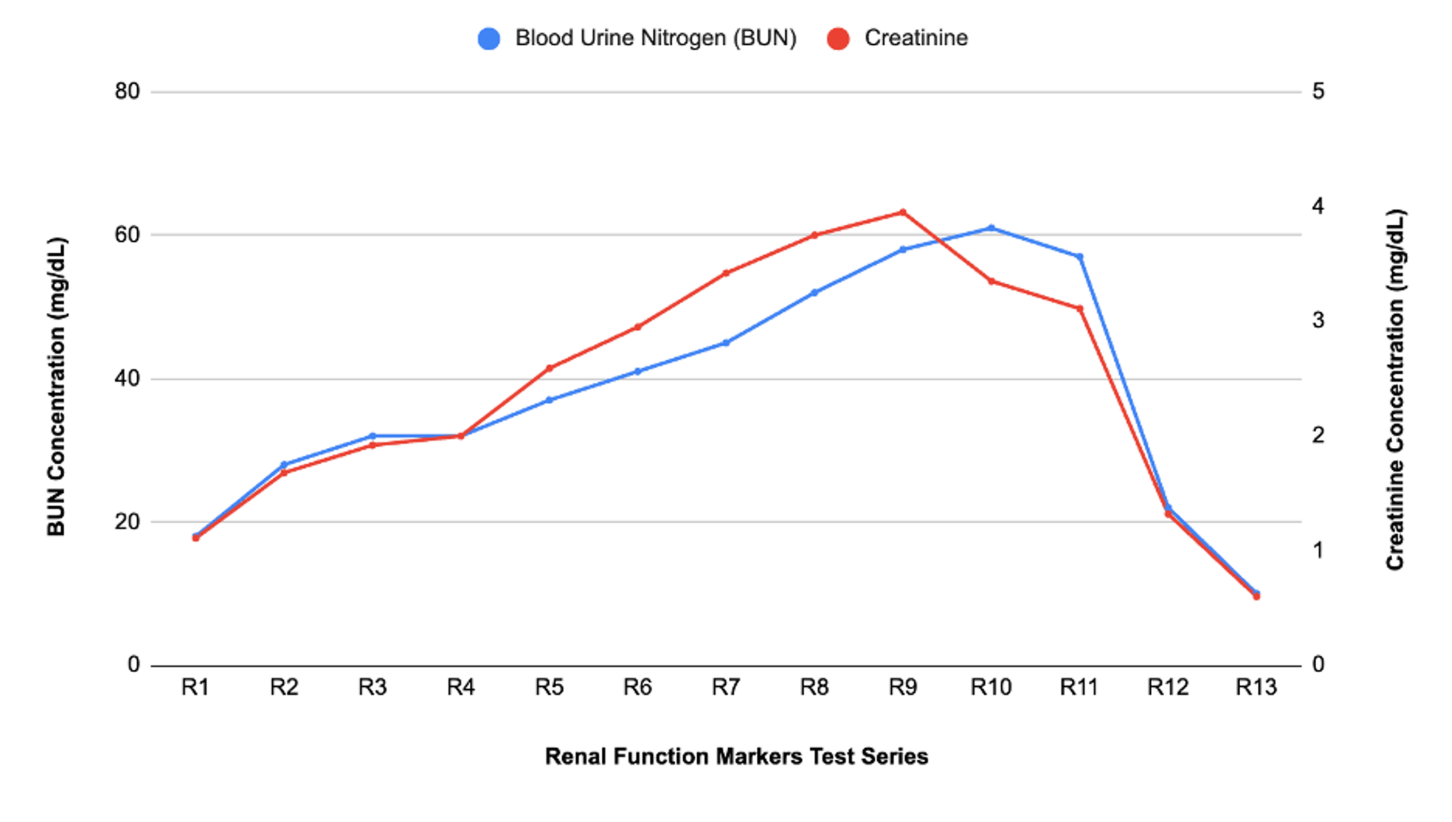
Patient’s renal function trend during hospitalization and the postpartum period. Values represent BUN and creatinine concentrations, labeled R1 through R13. R1–R11 were obtained during hospitalization. R12 and R13 were obtained during outpatient postpartum follow-up visits at 10 and 52 days postoperatively, respectively. BUN: blood urea nitrogen.

Nephrology was also consulted due to the patient’s elevated blood urea nitrogen (BUN), creatinine, and oliguria. BUN and creatinine were 37 mg/dL and 2.59 mg/dL, respectively, prompting the consultation. The patient received 80 mg of IV Lasix per Nephrology's recommendation. After receiving Lasix, the patient began diuresing approximately eight hours postoperatively. The trend of the patient’s urine output is shown in Figure 3, and the trend of the patient's BUN and creatinine is shown in Figure 4. HELLP syndrome was considered unlikely due to normal blood pressure, normal platelet count, and only mildly elevated liver function tests (LFTs). Nephrology, assuming atypical HELLP, ordered the patient to be given magnesium sulfate. Workup for infectious or autoimmune causes was negative.

**Figure 5:**
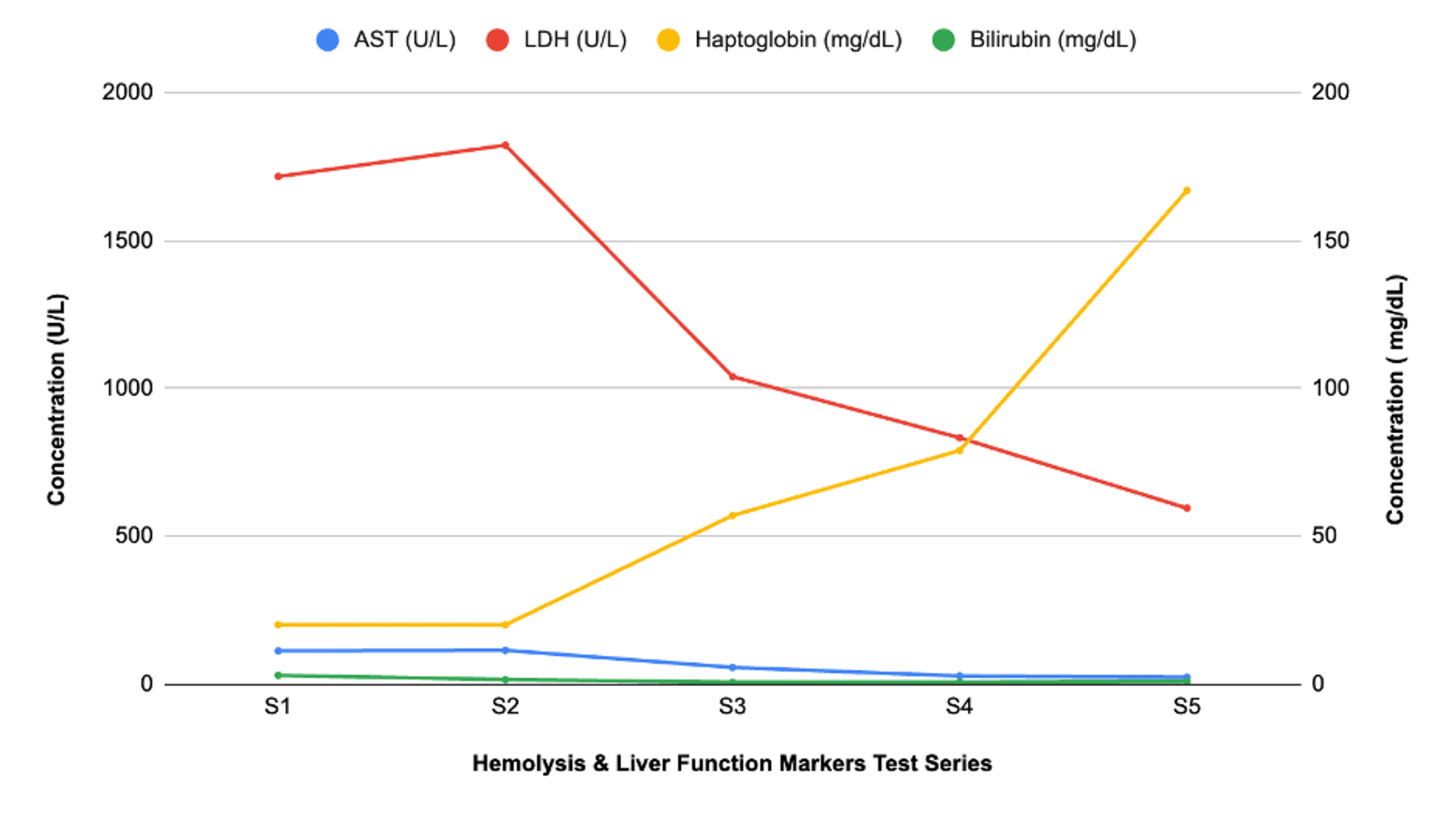
Patient’s hemolysis and liver function marker trends during hospitalization. Values represent AST, LDH, haptoglobin, and bilirubin concentrations labeled S1 through S5. All values were obtained during the patient’s hospitalization. AST: aspartate aminotransferase; LDH: lactate dehydrogenase.

**Figure 6:**
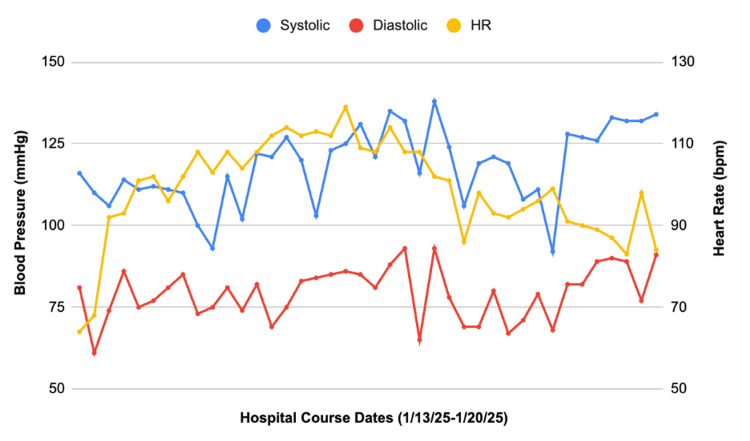
Trend in the patient’s blood pressure and heart rate recordings from the day of hospital admission to discharge. Multiple measurements were taken throughout each day. The graph emphasizes the stability in the patient’s vitals during her hospital course with no clinically concerning deviations.

A hematology/oncology consult noted multifactorial anemia likely due to delivery, hematoma, and potential hemolysis. Hemolysis labs were positive, with haptoglobin <20, LDH of 1823, total bilirubin of 2.9, and an elevated reticulocyte count. The trend of the hemolysis labs is shown in Figure 5. Although the direct antiglobulin test (DAT) was negative, it had been completed before the onset of anemia. HELLP and thrombotic microangiopathy (TMA) were considered unlikely due to normal platelets and the absence of schistocytes on the peripheral smear. Medication review, including oxytocin, terbutaline, cefazolin, azithromycin, metronidazole, and misoprostol, revealed no agents likely to cause hemolysis. An important point to note was that the patient never had hypotension, hypertension, or tachycardia, emphasizing the ambiguity of the case. The patient’s vitals throughout her hospital course are shown in Figure 6.

After hospital discharge, our patient presented to the clinic for her routine postoperative visit on postoperative day 10. She continued to have a subcutaneous hematoma, and a 0.5 cm superficial opening was noted on the right side of the incision, from which old blood was expressed. A repeat CBC and CMP were obtained at this visit for further monitoring. Our patient also returned on postoperative day 14. At that time, the incision was noted to be clean, dry, and intact, with positive induration but no discharge expressed upon palpation. On postoperative day 17, she was re-evaluated, and the findings remained unchanged, with the incision continuing to be clean, dry, and intact with persistent induration and no discharge expressed on pressure application. At her routine postpartum visit, the patient was clinically stable. CBC and CMP were within normal limits, and no further concerns were noted. She continued to follow up with nephrology to monitor her renal function, which had steadily improved since discharge. At her six-week postpartum visit, her BUN was 10 mg/dL (normal range: 6-20 mg/dL) and creatinine was 0.60 mg/dL (normal range: 0.57-1.00 mg/dL), both falling within normal limits.

## Discussion

Hemolysis is defined as the destruction of red blood cells, leading to the release of hemoglobin into the bloodstream. This process can lead to a cascade of complications, including AKI [5]. In our case, laboratory findings such as elevated LDH, low haptoglobin, elevated bilirubin, and a drop in hemoglobin were consistent with hemolysis. Our patient also exhibited elevated creatinine and oliguria, which were concerning for AKI.

The most common causes of hemolysis are diverse and can be broadly categorized into intrinsic and extrinsic factors. Intrinsic factors include structural abnormalities of red blood cells, such as sickle cell disease, while extrinsic factors include autoimmune conditions such as autoimmune hemolytic anemia, infections, and medications like heparin. In the postpartum period, hemolysis can be triggered by factors such as vascular injury, infection, and HELLP syndrome [6]. Despite an extensive workup, no definitive etiology for hemolysis was identified in our patient, highlighting the diagnostic challenges in the postpartum period.

AKI is the inability of the kidneys to effectively filter waste and regulate fluid balance, and it was also noted in this case. AKI can result from prerenal causes such as hypoperfusion, intrinsic injury such as acute tubular necrosis, or postrenal obstruction [7]. In the context of hemolysis, free hemoglobin released from used red blood cells can be directly toxic to renal tubular epithelial cells and may obstruct the renal tubules, resulting in acute tubular necrosis (ATN) [8]. This is a well-recognized mechanism of intrinsic AKI following hemolysis and may explain our patient’s clinical course of oliguria and elevated creatinine.

Early recognition of hemolysis and AKI is essential for timely management and preventing further complications. Prompt intervention, such as administering Lasix to reduce fluid overload, can help prevent the worsening of kidney injury. Effective monitoring and early treatment strategies are critical for improving patient outcomes. The management of hemolysis and AKI in the postpartum period requires a multifaceted approach. In this case, the patient’s hemolysis was managed with transfusions and fibrinogen administration, with close monitoring of laboratory parameters. A multidisciplinary team, including MFM, nephrology, and hematology, thoroughly investigated potential causes, including infection, HELLP syndrome, and hemolytic anemia.

Despite extensive workup, the exact cause of our patient’s hemolysis remained unclear, highlighting the importance of recognizing idiopathic hemolysis as a potential diagnosis in similar cases. While the hemolysis may have been triggered by the stress of an emergency C-section and the associated blood loss, other factors, such as vascular injury, tissue trauma, or minor infection, could have contributed to the patient's condition. Although AFE cannot be ruled out in this scenario due to the patient's sudden onset of clinical deterioration, the lack of a confirmatory diagnosis for AFE raises a stronger suspicion for idiopathic intravascular hemolysis. The uncertainty emphasizes the need for further research into idiopathic hemolysis in the postpartum period. By better understanding the triggers of pregnancy-related hemolysis, clinicians will be better equipped to manage these complex situations.

## Conclusions

Postpartum hemolysis and acute renal injury, though rare, can be life-threatening complications. Early recognition, appropriate diagnostic workup, and prompt multidisciplinary intervention are essential for preventing significant maternal morbidity. This case highlights the complex management of a postpartum patient who developed acute hemolysis and acute renal injury following an emergent C-section due to persistent fetal bradycardia. While the exact etiology of the hemolysis is unknown, it is likely multifactorial in this patient presentation. The patient's condition was effectively managed through transfusions, administering fibrinogen, and closely monitoring laboratory parameters. The consultation of a multidisciplinary team, including maternal-fetal medicine, nephrology, and hematology, was pivotal in diagnosing and managing the hemolysis, ensuring timely intervention and appropriate supportive care. Notably, the patient’s laboratory parameters, including LDH, haptoglobin, bilirubin, and creatinine levels, returned to normal following intervention, demonstrating the efficacy of comprehensive care. Further research exploring the potential underlying causes of pregnancy-related hemolysis, particularly in idiopathic cases, is essential for developing a more strategic approach to clinical management.
